# Fine Mapping and Identification of *SmAPRR2* Regulating Rind Color in Eggplant (*Solanum melongena* L.)

**DOI:** 10.3390/ijms24043059

**Published:** 2023-02-04

**Authors:** Huarong Fang, Peng Wang, Wanhao Wang, Jiechun Peng, Jieming Zheng, Guangwei Zhu, Chuan Zhong, Wenjin Yu

**Affiliations:** College of Agriculture, Guangxi University, Nanning 530004, China

**Keywords:** *APRR2*, BSA, chlorophyll, eggplant, premature termination codon, rind color

## Abstract

Rind color is an economically important agronomic trait in eggplant that impacts consumer preferences. In this study, bulked segregant analysis and competitive allele-specific PCR were employed to identify the candidate gene for eggplant rind color through constructing a 2794 F_2_ population generated from a cross between “BL01” (green pericarp) and “B1” (white pericarp). Genetic analysis of rind color revealed that a single dominant gene controls green color of eggplant peel. Pigment content measurement and cytological observations demonstrated that chlorophyll content and chloroplast number in BL01 were higher than in B1. A candidate gene (EGP19168.1) was fine-mapped to a 20.36 Kb interval on chromosome 8, which was predicted to encode the two-component response regulator-like protein Arabidopsis pseudo-response regulator2 (*APRR2*). Subsequently, allelic sequence analysis revealed that a SNP deletion (ACT→AT) in white-skinned eggplant led to a premature termination codon. Genotypic validation of 113 breeding lines using the Indel marker closely linked to *SmAPRR2* could predict the skin color (green/white) trait with an accuracy of 92.9%. This study will be valuable for molecular marker-assisted selection in eggplant breeding and provides theoretical foundation for analyzing the formation mechanism of eggplant peel color.

## 1. Introduction

Eggplant is an economically important vegetable crop worldwide, ranking with tomato and pepper as the three major vegetables of the Solanaceae family. The global eggplant planting area in 2020 was 1.846 million hectares, and its output increased to 56.302 million tons (data available at http://faostat.fao.org/ accessed on 13 April 2022). The eggplant rind color is a key factor that affects consumer choice directly, and breeders pay considerable attention to this trait. In the present market, the purple and green skin colors in eggplant are the most common, whereas white eggplant is a rarer type [1]. Two main pigments, anthocyanin and chlorophyll, determine the fruit color. Purple-skinned eggplants are rich in anthocyanin, green peel eggplant cultivars have a much higher chlorophyll content, while the pericarp of white eggplants contains little to no pigments [2].

Chlorophyll is responsible for capturing light energy, and its large accumulation is the main reason for the green tissue color [3]. In vegetative green tissues, chloroplasts are the site where chlorophyll biosynthesis takes place, used in photosynthesis [4,5]. Chlorophyll biosynthesis is a complex process involving a series of enzymes [6,7]. The mutation of any enzyme gene may lead to defects in chlorophyll synthesis and chloroplast development, resulting in varying changes in chlorophyll content in the pericarp [8].

Recently, progress has been made concerning the genetic analysis and gene mapping of eggplant rind color traits. Previous inheritance studies showed that purple color is dominant over other non-purple colors, and green is dominant over white [9]. The anthocyanin synthesis pathway in purple-colored eggplant fruits has been adequately studied. It involves three types of regulatory genes, fifteen structural genes, and many key enzymes to co-regulate anthocyanin synthesis [10,11,12,13]. The loci associated with anthocyanin pigmentation in eggplant fruits were mapped on chromosomes E05, E06, and E10, of which E10 had a remarkably high LOD value [14]. However, gene fine mapping of green/white color in eggplant peel has been less studied than purple. In the early 20th century, Tatebe [15,16] first reported six genes controlling color formation in eggplant fruits, namely C, P, D, G, Gv, and Puc, where G and Gv are genetic factors controlling chlorophyll formation during the pre-fruit development. Many years later, Doganlar et al. [17] found two *fc* quantitative trait *loci* (QTLs) related to eggplant fruit color (green or purple) on linkage groups 8 and 10, and our result was consistent with the previous finding.

Much effort has been dedicated to understanding the regulation of rind color in horticultural plants, and some QTLs and genes regulating chlorophyll accumulation and chloroplast development have been reported. In immature pepper fruits, Arnon et al. [18] identified two major QTLs, pc8.1 and pc10.1, controlling chlorophyll levels. *Pc8.1* had the most significant effect on chlorophyll content while *pc10.1* encoded a *Golden2*-like transcription factor (*GLK2*) and regulated chloroplast structure in early fruit development [19]. Numerous studies have shown that *GLKs* are essential transcription factors for chlorophyll biosynthesis, including *GLK1* and *GLK2* [20]. Correspondingly, transcription factors with similar functions include *TKN2*, *TKN4*, and *APRR2*, which have been reported in tomatoes [21,22]. *APRR2*, a gene involved in chlorophyll biosynthesis, has been identified in many plant species, especially in Cucurbitaceae. In cucumber, *APRR2* has been identified as a candidate gene for green pericarp, and its allele *aprr2* controls white pericarp [23,24]. Based on this finding, Yang et al. [25] found that the Csa3G904140 gene on chromosome 3 was related to white and green pericarp of cucumber, which was completely consistent with the *APRR2* location in the former study. In melon and watermelon, Oren et al. [26] confirmed the key role of the *APRR2* transcription factor underlying fruit color variation, showing that *APRR2* alleles generate the qualitative variation between dark and light green rind in both plants, and the *APRR2* expression was associated with the intensity of pigment. In *Wax Gourd*, Ma et al. [27] reported that *BhAPRR2* is involved in the regulation of peel color. A frameshift two-base deletion in this gene led to the obstruction of chloroplast development and chlorophyll synthesis in green-skinned wax gourds and resulted in the formation of the white peel variety. In *Zucchini*, a major QTL controlling the dark green color of immature and ripe fruit skins was identified, and two genes associated with *Arabidopsis APRR2*-like were located within these QTLs [28]. Their study also revealed that these two *APRR2*-like genes in the Cucurbitaceae family had termination codon mutations, resulting in color diversity associated with different chlorophyll content. Hence, the progress regarding the genetic control of fruit color in plants with close relatedness can provide an important reference for the genetic research in eggplant.

In the present study, we investigated the inheritance of eggplant rind color in F_1_, F_2,_ and BC_1_ populations derived from “BL01” (green) and “B1” (white) parental lines. Bulked segregant analysis sequencing (BSA-seq) was used to preliminarily map a candidate region for rind color in the F_2_ population, and molecular markers were developed to screen the recombinants and narrow down the initial region to achieve fine localization. A strong candidate gene *SmAPRR2* located within the fine-mapping interval was verified by sequence alignment and qRT-PCR analysis. This research has important applications for molecular maker-assisted selection (MAS) of green/white fruit eggplant and the scientific significance of exploring the formation mechanism of this trait, which further lays a theoretical foundation for analyzing the generation of abundant peel color in eggplant.

## 2. Results

### 2.1. Inheritance and Phenotypic Characterization of Rind Color in Eggplant

**Figure 1:**
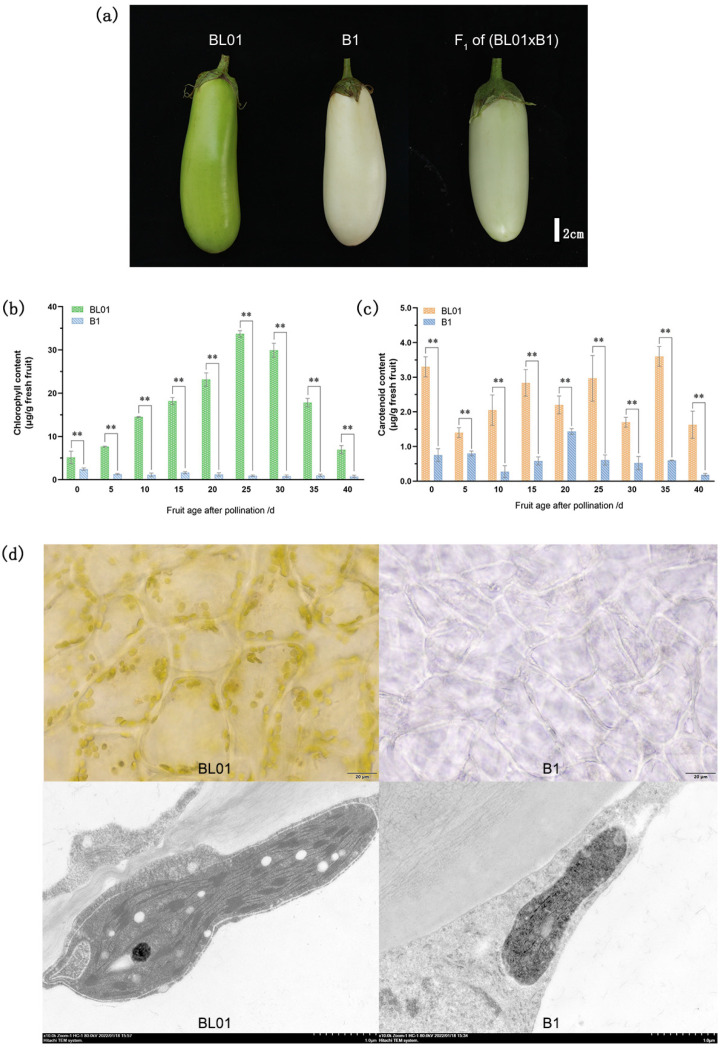
Phenotypic characteristics, pigment content, and cytological observations of the parental lines peel. (**a**) Peel characteristics of BL01 and B1, and their F_1_ progeny, Bar = 2 cm; (**b**) chlorophyll content and (**c**) carotenoid content of the parental lines peel; (**d**) cytological observations of the parents, from left to right, are BL01, and B1, from top to bottom, are fluorescence microscopy and transmission electron microscopy. **, *p* < 0.01.

The parental lines used in our study had clearly varied fruit skin hues (Figure 1a). BL01 has a green peel, while B1 has a white peel. In the F_1_ population, all the fruits showed a light green-skinned phenotype. The 2794 individuals in the F_2_ population were separated into 2094 green and 700 white peel eggplants, corresponding to a ratio of 3:1 (*χ* = 0.004, *p* = 0.948). All 44 individuals of BC_1_P_1_ had green fruit peels. BC_1_P_2_ populations had 21 with green pericarp and 20 with white pericarp, resulting in a ratio of 1:1 (*χ^2^* = 0.024, *p* = 0.876). The genetic analysis is shown in Table 1.

### 2.2. Determination of Pigment Contents

The chlorophyll and carotenoid content of parental lines’ peel were determined at different developmental stages (0, 5, 10, 15, 20, 25, 30, 35, 40 days after pollination, DAP). The pericarp of BL01 contained higher chlorophyll content than that of B1 at each testing times, with differences being highly significant (Figure 1b). Chlorophyll content in the green peel was the highest at 25 DAP, ~36.6 times higher compared to the white peel, which was consistent with the visual observations. In addition, the carotenoid content in BL01 rind was significantly higher than in B1 (Figure 1c). The above results indicated that more pigments accumulated in BL01 fruit skin, prompting us to further ascertain the differences in chloroplasts between BL01 and B1 cells.

### 2.3. Chloroplast Microscopic Observation

Eggplant pericarps were sliced into microsections and observed under a fluorescent microscope. The chloroplasts were densely distributed in BL01, and the visual field of its peel appeared green. Conversely, no chloroplasts were observed in B1, in accordance with the peel appearing white (Figure 1d). Therefore, the microscopic findings were in line with naked-eye observation and quantitative analysis of chlorophyll content. Through a transmission electron microscopy (TEM) assay, we observed that the chloroplasts of B1 exhibited premature senescence and a simple internal structure. Moreover, the grana thylakoids in chloroplasts of B1 were less numerous than in BL01 (Figure 1d).

### 2.4. Candidate Gene Located on Chromosome 8 Identified by BSA-seq

**Figure 2:**
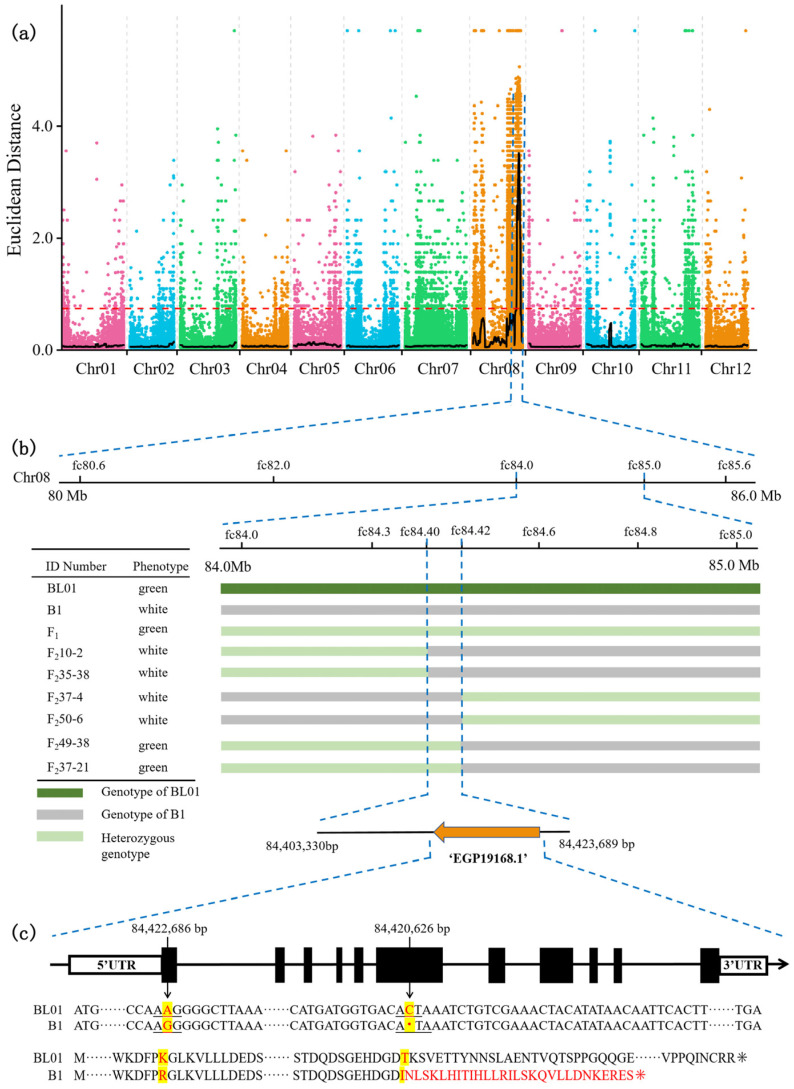
Mapping and cloning of the *SmAPRR2* gene in eggplant. (**a**) Manhattan plot of eggplant peel color mapping across BL01 and B1. (**b**) Fine mapping of the candidate gene. *SmAPRR2* gene was localized within a 20.36 Kb region between the flanking markers fc84.40 and fc84.42. (**c**) *SmAPRR2* gene structure and comparison of coding sequence and protein sequence between the parental lines (BL01 and B1). White boxes, black rectangles, and solid lines represent 5′ and 3′ UTR, exons, and introns, respectively. 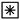 represent stop codon.

With bulked segregant analysis (BSA), 70,694,232 and 72,234,133 clean reads were obtained from BL01 and B1 parental lines, while 128,307,275 and 100,566,598 clean reads were obtained from the green-rind pool and the white-rind pool, respectively. The Q30 mean value reached 95.12, and the GC content range was 36.53~37.17%. After aligning the parent and F_2_ samples to the “guiqie1” reference genome, the average mapping rate was 97.16%. The average genome coverage depth was approximately 21.75×, and the genome coverage rate was about 98.69% (at least 1× coverage). Quality control suggested that the sequencing data can be utilized for subsequent mutation detection and correlation analysis. A total of 1,833,600 single-nucleotide polymorphisms (SNPs) were obtained from the four pools, including 481,492 high-quality SNPs. Additionally, these high-quality SNPs were used to calculate the SNP index between the two F_2_ bulks. Preliminary BSA-seq mapping results were obtained with the Euclidean distance (ED) and ΔSNP-index association algorithms. A confidence interval was found on chromosome 8 to be associated with the rind color trait, located within the 80.33~85.93 Mb region, spanning 5.60 Mb (Figure 2a). This region included 368 genes, 81 of which were found to have non-synonymous mutations by SNP analysis. Therefore, additional markers were developed on Chr8 to further pinpoint genes linked with the rind color trait in eggplant.

### 2.5. Fine Mapping and MAS Application Strategy of SmAPRR2

To further narrow the candidate interval, kompetitive allele-specifc PCR (KASP) makers were developed within the 5.60 Mb obtained from the initial localization. 2794 F_2_ individuals were analyzed using five KASP markers, narrowing the range to 83,970,554~84,848,065 bp with a physical distance of roughly 877.5 Kb. A total of 40 recombinant plants were selected from this population. Subsequently, based on the whole genome sequencing data of the parental lines, new KASP and Indel (base differences ≥3 bp) markers between the flanking markers fc84.0 and fc85.0 at 0.2~0.3 Mb intervals were selected for primer design. Two polymorphic Indel markers (fc84.3 and fc84.6) were developed to genotype these recombinant plants, and the mapping range was narrowed again to 369.6 Kb between 84,307,771 bp and 84,677,370 bp. Other Indel markers and cleaved amplified polymorphic sequence (CAPS) markers were designed for this candidate region, from which two polymorphic markers were discovered and applied for genotyping the recombinant plants. Eventually, the candidate gene regulating pericarp green/white color was localized between the Indel marker fc84.40 and the CAPS marker fc84.42 within a 20.36 Kb region (Figure 2b). This interval only contained a strong candidate gene, EGP19168.1, which was annotated as *Solanum melongena APRR2* (designated as *SmAPRR2*), flanked by two and four recombinant plants, respectively.

**Figure 3:**
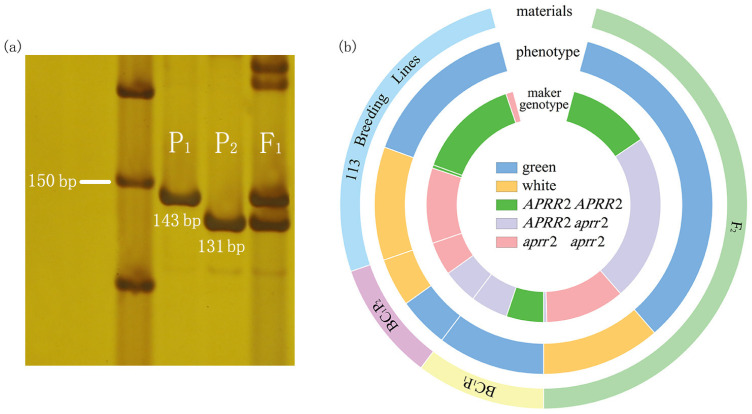
Segregation analysis of Indel marker fc84.40. (**a**) PCR amplification results of fc84.40 marker in P_1_, P_2,_ and F_1_. (**b**) The verification of fc84.40 marker in two backcrossing populations, 2794 F_2_ populations, and 113 breeding lines. The outer circle indicates the populations used for segregation analysis; the middle circle indicates the phenotype of the materials; the inner circle indicates the maker genotype of the materials.

The Indel marker fc84.40, located 14.3 Kb from the *SmAPRR2* gene, was initially used to verify the consistency of genotype and fruit color phenotype in two BC_1_ populations and a 2794 F_2_ population. Genotypes and phenotypes matched perfectly in the two BC_1_ populations, and just two recombinants were found in the F_2_ population. Then, the fc84.40 marker was used to screen 113 breeding lines, among which 66 lines had a green peel and 47 lines had a white peel (Supplementary Table S3). As shown in Figure 3b, the verified accuracy rate was 92.9% in breeding lines, with six green lines and two white lines showing mismatched genotypes and phenotypes. We subsequently resequenced these (unpublished) germplasm resources at a depth of 10× and performed Sanger sequencing of the *SmAPRR2* gene. We found six mismatched green lines consistent with the BL01 sequence. Therefore, we speculated that the mismatch between the marker genotypes and phenotypes of these six resources might be due to the fact that fc84.40 was 14.3 Kb away from *SmAPRR2*, which was a closely linked marker and did not reach the degree of co-separation. Moreover, the sequencing results of two mismatched white lines showed that the key SNP deletion (ACT→AT) site leading to early codon termination did not change in the *SmAPRR2* of these two materials, whose coding sequences were the same as that of BL01. The spatio-temporal expression results of *SmAPRR2* also showed no significant difference from BL01. Thus, we speculated that the peel color of these two white eggplant lines may be controlled by other independent genes. In addition, in the remaining 105 inbred lines that matched the marker genotype and phenotype, resequencing revealed that the *SmAPRR2* gene had one or more amino acid mutations that were abundant and irregular between the green lines, as well as between the white lines.

In conclusion, Indel marker fc84.40 was considered to be a fast, effective, and economical method for the identification of green/white peel traits in eggplant, and Sanger sequencing can be used for further accurate identification.

### 2.6. Sequence Alignment and Expression Analysis of the SmAPRR2 Candidate Gene

To analyze the *SmAPRR2* gene sequence, we designed primers to amplify its full-length coding sequence (CDS) from both parents and performed gene cloning. Sequencing results were aligned using DNAMAN v.9 (Lynnon Biosoft, USA) and were listed in Supplementary Table S4. The *SmAPRR2* CDS region was 1674 bp, with 12 exons. There was a C base deletion (ACT→AT) in the 6^th^ exon of B1, leading to an earlier stop codon, which resulted in a 296 amino acid deletion compared with the protein sequence encoded by *SmAPRR2* in BL01 (Figure 2c). In addition, a nucleotide mutation (G→A) was present in the 1st exon of BL01, which caused an amino acid substitution (R→K).

**Figure 4:**
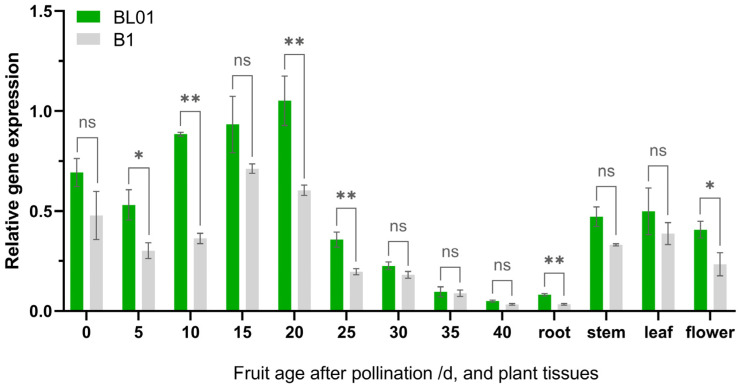
*SmAPRR2* expression analysis. Expression analysis of *SmAPRR2* in fruit peel and different issues of BL01 and B1. * 0.01 < *p* < 0.05; ** *p* < 0.01. “ns” means no significant difference. Green bars show the *SmAPRR2* expression of green-skinned eggplant (BL01) and gray bars show that of white-skinned eggplant (B1).

Moreover, the expression of the *SmAPRR2* was also analyzed with qRT-PCR in parental fruit rinds (0, 5, 10, 15, 20, 25, 30, 35, 40 DAP) and other tissues (including root, stem, leaf, and flower). The results revealed a large difference in *SmAPRR2* expression in pericarps and various tissues of BL01 and B1 (Figure 4). Regardless of the period of fruit development, the green peel had consistently higher *SmAPRR2* expression than the white peel. The *SmAPRR2* expression level in the BL01 peel (1.05) reached its peak at 20 DAP and was almost 1.74 times greater than in the B1 peel (0.60), with a significant difference. *SmAPRR2* expression in both green and white peel eggplant showed a downward trend from 0~5 DAP, gradually increased from 5~20 DAP, and finally decreased after reaching the peak. The expression levels of *SmAPRR2* in the parents were not significantly different in leaves and stems but had significant differences in roots and flowers.

### 2.7. SmAPRR2 Protein Domains

**Figure 5:**
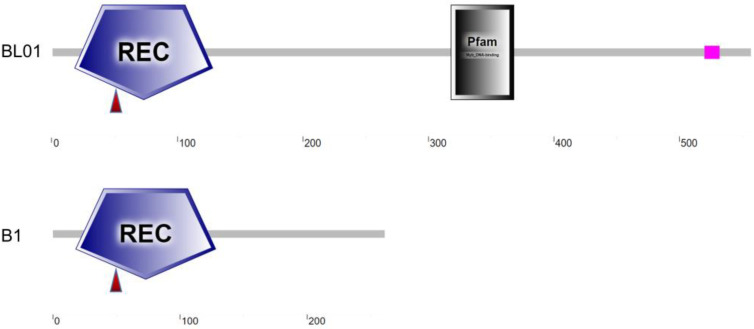
*SmAPRR2* protein structure. The red triangle indicates one amino acid substitution occurring in the REC domain of BL01 and B1; the MYB-like DNA-binding domain is absent in B1.

According to SMART (https://smart.embl-heidelberg.de/ accessed on 12 June 2022) analysis, the *SmAPRR2* protein structure in BL01 contained a REC domain (18th~128th amino acids) and an MYB-like DNA-binding domain (318th~368th amino acids). On the other hand, the *SmAPRR2* allele in B1 only contained a REC domain. A non-synonymous mutation occurred in the REC domain resulting in amino acid substitutions, and a premature termination codon in B1 resulted in the lack of an MYB-like DNA-binding domain (Figure 5), which was reported to be responsible for fruit color [29].

### 2.8. SmAPRR2 Protein Phylogenetic Analysis

**Figure 6:**
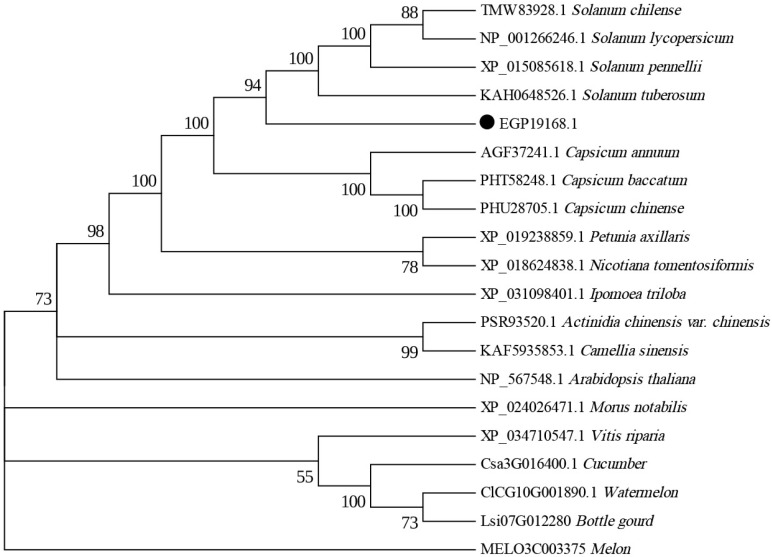
Phylogenetic analysis. Phylogenetic tree of *SmAPRR2* and its homologous proteins. Numbers at the tree forks indicate bootstrap values.

A phylogenetic analysis was performed to further investigate the relationship between the *SmAPRR2* protein and its homologous sequences. After downloading high-level homologous protein sequences in FASTA format using NCBI BLAST (NCBI, Bethesda, MD, USA), a phylogenetic tree with 1000 bootstrap repetitions was built in MEGA 6.0 software using the bootstrap method. The neighbor-joining tree revealed that EGP19168.1 (*SmAPRR2*) has a close phylogenetic relationship with plants of the Solanaceae family (*Solanum chilense*, *Solanum lycopersicum*, *Solanum pennellii*, and *Solanum tuberosum*), which were located on the same branch (Figure 6). This indicated that the *SmAPRR2* gene is evolutionarily conserved in the Solanaceae family.

## 3. Discussion

Eggplant peel color is one of the crucial appearance qualities and an important characteristic affecting its merchantability, so it has become a key trait for breeders to focus on. Anthocyanin and chlorophyll content are the main factors influencing the color of eggplant fruit peel. At present, most of the eggplants we can see in the market are purple or green peel, while white eggplant fruits are relatively uncommon [1]. Halsted [30] was the first to attempt an analysis of the inheritance of eggplant fruit color. Subsequently, some studies on the skin color of Solanaceae crops reported similar genetic patterns, with green being dominant over white [31,32]. Likewise, in our study, the phenotype statistics were found to be consistent with a Mendelian single-gene segregation ratio, indicating that a single dominant gene controls the green color of eggplant peel. Although inheritance models have been extensively proposed for eggplant fruit color, little is known about the underlying genetic mechanism of green and white rind color [31]. Therefore, identifying the candidate gene that regulates the green/white color of eggplant pericarp is valuable to further study the regulatory mechanisms of abundant peel color in eggplant.

Gene mapping and molecular marker-assisted selection have become routine methods for genetically improving many crops [33]. In our study, we employed BSA-seq and mapped the locus controlling green/white rind color to a 20.36 Kb interval on chromosome 8 using Indel and CAPS markers. Only the *SmAPRR2* gene was located within this region, predicted to encode a two-component response regulator-like protein by annotation. The corresponding orthologous gene in *Arabidopsis* is an *APRR2*-like gene, a member of the *APRR* family. Interestingly, *APRRs* in *Arabidopsis* were earlier reported to be involved in circadian rhythm regulation [34]. However, *APRR2*-like genes in recent years have been further identified as key transcription factors involved in the regulation of plastid metabolism and therefore influencing color development in various species [23,24,35]. Our study supports those previous findings based on the disparities observed between green and white-colored eggplant parents regarding plastid structure and rind color. Cytological observation revealed that the number of chloroplasts and thylakoids in the white peel parent was lower than the green peel parent. Rind color is also affected by the presence or the absence of pigment located in the subepidermal cell layers [3]. The *APRR2*-like gene was shown to be correlated with pigment accumulation [26]. Notably, overexpression of the *APRR2* gene in tomato increased chlorophyll and carotenoid content [21]. Here, we measured chlorophyll and carotenoids in the pericarp and found that their contents were higher in green peel eggplants than in white peel ones. The chlorophyll content in BL01 increased in the early stages of growth and development and steadily declined after peaking (nearly 25 DAP). In contrast, the chlorophyll content in B1 remained stable and was only detected in trace amounts. This trend was in agreement with the results of the visual observations. An explanation for the above might be that a negative balance between chlorophyll synthesis and catabolism drives its degradation during fruit development, leading to the green color fading off in the fruit skin [36]. On the other hand, Yamauchi et al. [37] showed that peroxidase is involved in chlorophyll degradation, during which peroxidase oxidizes the phenolic compounds and forms phenoxy radicals. The phenoxy radicals oxidize Chl and its derivatives to produce colorless low molecular weight compounds. The above suggested that the green/white color of eggplant rind correlates with the chloroplast development regulated by the *SmAPRR2*.

The *SmAPRR2* sequence alignment between the two parental lines revealed that a single-base deletion generates prematurely terminated translation in white-skinned eggplant, resulting in color difference from the green-skinned eggplant ultimately. Likewise, early codon termination in *Cucumis APRR2* disrupted chlorophyll synthesis, which caused the pericarp to change from green to white, so the gene was considered to be critical in controlling cucumber fruit color [23,38]. Pan [21] also provided evidence for an association between a null mutation in the *APRR2* gene and fruit color intensity in green peppers. A search for conserved domains revealed that *SmAPRR2* carries an MYB-like DNA-binding domain, primarily responsible for fruit color [29]. Thus, the absence of the domain in white eggplant due to premature termination codon can also account for its pericarp color difference compared to green eggplant. Furthermore, qRT-PCR analysis indicated that *SmAPRR2* had a higher expression level in the green peel than in the white peel at the fruit developmental stage from 0 DAP to 40 DAP. In green-skinned eggplant, the transcription factor reached a peak expression at nearly 20 DAP and then was gradually downregulated. This pattern was consistent with previous reports on *APRR2* expression in other crops. The *APRR2* expression in green-skinned cucumbers tended to be at the highest level in 12 DAP and then gradually decreased [23]. In melon, *CmAPRR2* peak expression in the peel occurred at ~15 DAP before the fruit started to ripen and change color. In addition, the expression peak of *SmAPRR2* was slightly earlier than the time when the green color of the pericarp reached the deepest intensity. The *SmAPRR2* exhibited higher expression in green tissues (leave and stem), which further reinforces that the *SmAPRR2* gene function is related to photosynthesis [3].

According to the findings, we used Indel marker fc84.40 at a distance of 14.3 Kb from the *SmAPRR2* gene, for preliminary verification of genotype and phenotype in two BC_1_ and an F_2_ population. The results showed that the maker genotypes and phenotypes of BC_1_ plants were fully matched, and only two recombinant plants were screened in the F_2_ population. Then, 113 eggplant germplasms were identified using the fc84.40 marker, and the genotype–phenotype match rate was 92.9%. We have also tried to design a CAPS/dCAPS marker based on the key site (ACT→AT) on *SmAPRR2*, which needed high requirements for enzyme and experimental technique and had the defects of inefficiency, high cost, and inconvenient operation. However, choosing this Indel marker to detect breeding lines was not only simple and fast to perform, but also inexpensive, so the fc84.40 marker has great application in practical breeding work. Furthermore, we resequenced these (unpublished) 113 germplasms at 10× depth and Sanger sequenced the *SmAPRR2* gene. The results showed that there were 105 inbred lines with matching marker genotype and phenotype, and the *SmAPRR2* gene had single or multiple amino acid mutations, which were abundant and irregular between the green lines as well as between the white lines. The six mismatched green lines were consistent with the BL01 sequence. Thus, we speculated that the marker genotype–phenotype mismatch in these six germplasms was most likely because fc84.40 was 14.3 Kb away from *SmAPRR2*, and it is a tightly linked marker without reaching co-segregation. Moreover, the *SmAPRR2* sequencing results of two mismatched white lines showed that the critical site did not change, and their coding sequence was the same as BL01. Then, spatio-temporal expression analysis of *SmAPRR2* in two mismatched white lines also showed no significant difference from BL01. We thus inferred the existence of other separate genes controlling green/white fruit color in eggplant. In subsequent studies, these two white lines can be used to cross with green germplasms to construct new populations and find additional genes regulating this trait, thereby enriching the eggplant fruit color genetic system.

In this paper, we used forward genetics to first fine-map the gene regulating the green/white color of eggplant peel. Combining with cytological observations and spatio-temporal expression analysis, we cloned and Sanger sequenced the *SmAPRR2* gene that affects pericarp color by regulating chloroplast development. Coincidentally, a dozen days after our article was preprinted (https://doi.org/10.21203/rs.3.rs-1884583/v1), a similar study by Arones et al. [39] closely followed us with a preprint (https://doi.org/10.1101/2022.08.23.504925). Through comparison, it is found that they constructed a MAGIC population to preliminarily locate candidate regions regulating green pigmentation in the eggplant peel by GWAS analysis, and then used the annotation information of the genes contained in the candidate intervals to directly predict *SmAPRR2*. Although this study did not narrow down the interval to one gene nor did it analyze expression for the predicted gene, it does provide proof of our result. We screened 113 breeding lines for resequencing and found that the *SmAPRR2* sequence had abundant and irregular one or more amino acid mutations among green lines as well as among white lines. We suggested that those mutations are not sufficient to affect the function of *SmAPRR2*, and the key locus that really causes peel green to white is the SNP deletion (ACT→AT) leading to the frameshift mutation. Based on the above result, we provided a scheme for verifying the fruit color trait in eggplant breeding work. Preliminary screening of germplasm collections can be performed using the tightly linked marker fc84.40 (accuracy of about 92.9%), which is fast, efficient, and economical; another way is to sequence the *SmAPRR2* gene in the germplasms, which can achieve further accurate identification of the genotype. In summary, our study illustrated the inheritance pattern of eggplant rind color (green/white) and firstly reported *SmAPRR2* as a strong candidate gene for regulating this trait by controlling chloroplast development. Our results promote molecular maker-assisted selection in eggplant breeding and facilitate the exploration of the underlying regulatory mechanisms controlling peel color.

## 4. Materials and Methods

### 4.1. Plant Materials and Phenotypic Evaluation

The eggplant inbred lines BL01 and B1 were selected as the male (P_l_) and female (P_2_) parents, respectively, to construct populations (F_1_, F_2_, and BC_l_) for the genetic analysis of green/white rind color. The skin color of BL01 fruit is green, while that of B1 fruit is white. F_1_ plants were obtained by crossing BL01 and B1, and the backcross populations were produced by hybridizing F_1_ plants with each parent to create BC_1_P_1_ and BC_1_P_2_, respectively. To identify candidate gene for pericarp color, an F_2_ population with 2794 individuals was constructed by self-pollinating the F_1_ plants. The phenotype of rind color was evaluated by visual observation, and eggplant fruits were categorized into green and white categories based on their appearance 25 days after pollination (DAP). All plants were grown in the field at Guangxi University under natural sunlight from summer 2020 to spring 2021.

### 4.2. DNA Extraction

Young leaves were collected and stored at −80 ℃ until their genomic DNA was extracted, using the cetyltrimethylammonium bromide (CTAB) method [40]. DNA quantification was carried out with an ultra-micro spectrophotometer (K5800, KAIAO, Beijing, China), and its quality was evaluated by electrophoresis in a 1.2% agarose gel.

### 4.3. Pigment Extraction and Measurement

The fruits were collected at different developmental stages (0, 5, 10, 15, 20, 25, 30, 35, and 40 DAP). Their pericarps were longitudinally cut into slices about 1 cm wide and 0.2 cm thick with a razor blade, and then ground into powder using liquid nitrogen. A 1.0 g sample powder was weighed and placed into a 15 mL centrifuge tube, and 10 mL of anhydrous ethanol was added to extract the pigments in the dark for 24 h. The absorbance values of chlorophyll a, chlorophyll b, and carotenoids at 665 nm, 649 nm, and 470 nm were measured by a microplate reader (Infinite M200, Männedorf, Switzerland), respectively. The pigment content was calculated from the following equations as described previously [41]:

C_a_ (mg/L) = 13.95 × A_665_ − 6.88 × A_649_, (1)

C_b_ (mg/L) = 24.96 × A_649_ − 7.32 × A_665_, (2)

C_x.c_ (mg/L) = (1000 × A_470_ − 2.05 × C_a_ − 114.8 × Cb)/245, (3)

Pigment content (μg/g) = [1000 × pigment concentration (mg/L) × extracted liquid product (L) × dilution factor]/sample weight (g),(4)

Total chlorophyll content (μg/g) = C_a_ content + C_b_ content, (5)

where C_a_ is the concentration of chlorophyll a, C_b_ is the concentration of chlorophyll b, and C_x.c_ is the carotenoid concentration. A_665_ is the absorbance of chlorophyll a at 665 nm, A_649_ is the absorbance of chlorophyll b at 649 nm, and A_470_ is the absorbance of carotenoid at 470 nm.

### 4.4. Cytological Observation

To observe the chloroplasts in the cells, pericarps from parental fruit at 25 DAP were excised with a sterile blade. The tissues were placed on a microslide with a tiny drop of distilled water, pressed onto a coverslip, and made into microscopic sections. The samples were observed and photographed under a fluorescent microscope (BX53, Olympus, Japan) at 400×.

Transmission electron microscopy (TEM) was carried out to observe the ultrastructure of chloroplasts in the 25 DAP fruit peel of the parental lines. The pericarps were sliced to a size of 1 mm^3^, and immediately placed in an electron microscope fixative solution for fixation; then, they were washed using 0.1 M phosphate buffer (PB, pH7.4) and post-fixed with 1% OsO4 in 0.1 M PB; dehydration at room temperature was followed. Subsequently, resin penetration, embedding and polymerization were carried out. Polymerized samples were trimmed into ultrathin sections, sliced to a thickness of 60~80 nm, and stained. Finally, the plastids were observed using an HT7800 (HITACHI, Hitachi, Japan) transmission electron microscope.

### 4.5. BSA-seq Mapping Approach

From 2794 F_2_ individuals, 60 plants with extreme phenotypes (30 with green peel and 30 with white peel color) were selected to construct two pools, one green-skinned pool and one white-skinned pool. Association analysis was performed for the two F_2_ pools and the parental pools, with “guiqie1” [42] as the reference genome. The pooled DNAs were used for DNA library preparation, and the qualified library was sequenced on the Illumina HiSeq™ PE150 platform (San Diego, CA, USA). Raw reads obtained by high-throughput sequencing were analyzed and converted into sequenced reads after base calling. Raw reads were filtered to obtain clean reads for subsequent sequencing analysis to ensure the quality of analysis. The obtained reads were compared with the “guiqie1” reference genome to perform variant detection. Two association analysis methods, the Euclidean distance (ED) and ΔSNP-index association algorithm, were used to identify region associated with the target trait.

### 4.6. Fine Mapping and Marker Development

To narrow the preliminary region, KASP markers were designed for each 1~2 Mb distance in this interval based on the BSA-seq data. The mixture prepared for PCR amplification was according to the manufacturer’s instructions (LGC Genomics, Shanghai, China). The volume of the PCR reaction was 3 μL, including 1.0 μL of DNA (8~15 ng μL^−1^), 1.5 μL of 2× master mix, and 0.5 μL of primer mix. Amplification was performed using landing PCR; the reaction conditions were as follows: heat treatment at 95 °C for 15 min; denaturation at 95 °C for 20 s, annealing and extension between 65 and 55 °C for 1 min, 10 cycles (each cycle reduced by 1.0 °C); and denaturation at 95 °C for 20 s, annealing and extension at 57 °C for 1 min, 26 cycles; then hold at 4 °C in the dark condition. After amplification, fluorescence scanning and genotyping were carried out. We initially designed 5 pairs of KASP markers to analyze 2794 F_2_ plants to identify recombinant plants (Supplementary Table S2). New markers were further developed within the flanking markers to detect the genotype of the recombinants, and the genotype–phenotype joint analysis was used to infer the most likely candidate region. The primers used are listed in Supplementary Tables S1 and S2.

### 4.7. Cloning and Sequencing of Candidate Gene

The coding sequence of candidate genes from “BL01” and “B1” was cloned. The primer sequences were designed with the Primer 5 software (Premier, Canada) based on the reference genome, shown in Supplementary Table S1. Total RNA from parental materials was extracted using a Plant RNA Purification Kit (Tiangen, Beijing, China), following the manufacturer’s instructions. The first strand of complementary DNA (cDNA) was synthesized using a 5× All-in-One Master Mix kit and an AccuRT Genomic DNA Removal kit (Diamed Life Sciences, Mississauga, ON, Canada). Using the 2×A8 FastHiFi PCR Master Mix (Aidlab, Beijing, China), PCR amplification was performed under the following conditions: initial denaturation at 94 °C for 5 min; followed by 35 cycles of denaturation at 94 °C for 20 s, annealing at 56 °C for 20 s, and extension at 72 °C for 1.5 min; a final extension step at 72 °C for 7 min. The PCR products were detected by electrophoresis on a 1.2% agarose gel, and the target bands were isolated and purified by gel cutting. Subsequently, we constructed the expression vector using the zero-background pTOPO-Blunt cloning kit (CV16) from Aidlab (Beijing, China), as instructed by the manufacturer: 1 μL of pTOPO-Blunt vector, 1 μL of 10× Enhancer, 6 μL of sterile water and 2 μL of PCR purified product were ligated at 37 °C for 10 min. The vectors were transferred into Trans5α chemically compatible cells following the manufacturer protocol (TransGen Biotech, Beijing, China). The proper PCR colony clones were selected and sent to Shanghai Shengong Biotechnology Co., Ltd. (Shanghai, China) for Sanger sequencing confirmation. The sequencing chromatograms were validated with multiple alignments using DNAMAN v.9 software (Lynnon Biosoft, San Ramon, CA, USA).

### 4.8. RNA Extraction and Gene Expression Analysis

Pericarp samples at different developmental stages (0, 5, 10, 15, 20, 25, 30, 35, 40 DAP) and various tissue samples (root, stem, leaf, flower) were collected from the parent plants. Total RNA from the above materials was extracted using the Plant Total RNA Purification Kit (Eastep^®^ Super, Beijing, China) according to the manufacturer’s instructions. cDNA was synthesized using the Reverse Transcriptase RT Master Mix (Takara, Kusatsu, Japan). The primer sequences for the candidate gene and the reference gene (ACTIN, EGP21177.1) were designed by Primer 5 software (Supplementary Table S1). To examine the candidate gene’s expression level, qRT-PCR analysis was performed in a QTOWER 2.2 qPCR instrument (Jena, Germany). A 20 μL reaction volume was used for the PCR amplification, which contained 2 μL of 100 ng cDNA, 0.8 μL of primer (10 μm), 10 μL of TB Green^®^ Premix Ex Taq II (2×; Tli RNaseH Plus; Takara Bio), 0.4 μL of ROX Reference Dye II (50×), and 6 μL of sterilized distilled water. Amplification and melt curve analysis were carried out as follows: heating at 95 °C for 5 s and 34 s at 60 °C for 40 cycles, high-resolution melting at 95 °C for 15 s, then 60 °C for 1 min and 95 ℃ for 15 s. The values of three reactions were averaged, and relative expression was determined using the 2^−ΔΔCt^ method [43].

## Figures and Tables

**Table 1:** Segregation of rind color in eggplant populations.

Population	No. Plants Tested	Green:White	Expected Mendelian Distribution	*χ^2^*	*p*
F_1_ ^a^	35	35:0	-	-	-
BC_1_P_1_ ^b^	44	44:0	-	-	-
BC_1_P_2_ ^c^	41	21:20	1:1	0.024	0.876
F_2_ ^d^	2794	2094:700	3:1	0.004	0.948

^a^ F_1_ = BL01 × B1; ^b^ BC_1_P_1_ = F_1_ (BL01 × B1) × BL01; ^c^ BC_1_P_2_ = F_1_ (BL01 × B1) × B1; ^d^ F_2_ population was derived from the self-pollination of F_1_ (BL01 × B1).

## Data Availability

The data presented in this study are available in this article and as Supplementary Materials.

## Supplementary Materials

The following supporting information can be downloaded at: https://www.mdpi.com/article/10.3390/ijms24043059/s1.
