# Supplementary material for: Fine Mapping and Identification of SmAPRR2 Regulating Rind Color in Eggplant (Solanum melongena L.)
Source: Int J Mol Sci. 2023 Feb 4;24(4):3059. doi: 10.3390/ijms24043059 (PMC9964064; doi:10.3390/ijms24043059)
Supplement: Supplementary file 1 [file ijms-24-03059-s001.zip › ijms-2188812-supplementary.pdf]

Title: Fine mapping and identification of *SmAPRR2* regulating rind color in eggplant (*Solanum melongena* L.)

Supplementary Information

Table S1 Sequences of primers used in the study

Primer ID	Annotation	Forward primer	Reverse Primer
EGP19168.1	CDS amplication	ATGATTTGCATTGAGG ATGAATTATTGG	TCATCTCCGACAATTGA TTTGTGGAGG
SmAPRR2	qRT-PCR analysis	CCGGTAAAAGAATCTC TCCT	TTTATCACAGCCCGATA ACT
ACTIN	qRT-PCR analysis	TATTGTGGGTCGTCCT CG	TCTCTCTGTTGGCCTTG G
fc84.3	Indel maker	TATGAAACTTAGGCGG TGGG	AATCGAACCCTCGACA CTTG
fc84.40	Indel maker	TTGAGCGGCAACAACA TAGC	GTACTCAGGTCCCCTTC CGAT
fc84.6	Indel maker	TGGTGAGTTCTTGTCA CCTG	TGTTTGAGGTGAGGTA CTGCTT
fc84.42	CAPS (*SspI*)	AGTCGGAGTAATTGAT AAATTTGTT	CTATGTGCAAGGTCTA GAAAAGAGT

Table S2 Primers for KASP markers

ID	primer_X	Primer_Y	Primer common
fc80.6	GAAGGTGACCAAGTT CATGCTGAATGAGACC CAGCACTTACATGT	GAAGGTCGGAGTCAA CGGATTGAATGAGACC CAGCACTTACATGA	GAGCTGGGCTTTGGA TATGTTGGC
fc82.0	GAAGGTGACCAAGTT CATGCTGGTTTACATC TGGACTCGTCTTTC	GAAGGTCGGAGTCAA CGGATTGGTTTACATC TGGACTCGTCTTTG	CACTGCCGACATAGAT GAATTACCAAG
fc84.0	GAAGGTGACCAAGTT CATGCTCATTTTCCTCT CTTCCACCGATGTA	GAAGGTCGGAGTCAA CGGATTTTTCCTCTCTT CCACCGATGTC	AAATTTAGTAGCTAAT TGTTGGGTATGTAAAT TC
fc84.8	GAAGGTGACCAAGTT CATGCTGTGTGTGCTT CTGTCACAACAC	GAAGGTCGGAGTCAA CGGATTGGTGTGTGCT TCTGTCACAACAT	TTACATAGCCCATAAA AGCATCTTAAACTAG
fc85.0	GAAGGTGACCAAGTT CATGCTGAGAGTTCAA GTCACATTGCAAGG	GAAGGTCGGAGTCAA CGGATTTGAGAGTTCA AGTCACATTGCAAGT	GCCGAAAAATCTTCA TTGGAACTTGATG
fc85.6	GAAGGTGACCAAGTT CATGCTGTAATTTGTCT	GAAGGTCGGAGTCAA CGGATTGTAATTTGTC	CTTTTCTTTAGGACTA ATTTATTTAGGCCTTT AATT

	AAGTCAAACTATATGA CATAT	TAAGTCAAACTATATG ACATAA

Table S3 Genotype identification of fruit rind color in the 113 eggplant germplasm resources

Number	Variety Name	Fruit color	Genotype
1	SmL544	green	green
2	SmL522	green	green
3	SmL151	green	green
4	SmL312	green	green
5	SmL31T	green	green
6	SmL1A	green	green
7	SmL111	green	green
8	SmLY11	green	green
9	SmL166	green	green
10	SmLCQD	green	green
11	SmLFZ11	green	green
12	SmLXD1	green	green
13	SmL118	green	green
14	SmL101	green	green
15	SmL137	green	green
16	SmL53cb	green	green
17	SmL708	green	green
18	SmLNY	green	green
19	SmL860	green	green
20	SmLG81	green	green
21	SmLG77	green	green
22	SmL233	green	green
23	SmL314	green	green
24	SmLG89	green	green
25	SmL342	green	white
26	SmL266	green	white
27	SmL211	green	white
28	SmL138	green	white
29	SmLZYQ02	green	white
30	SmLZYQ31	green	white
31	SmL119	green	green
32	SmL411	green	green
33	SmLSHF	green	green
34	SmLG1	green	green
35	SmLS152	green	green
36	SmLDT	green	green
37	SmLQ4	green	green
38	SmLY2	green	green

39	SmLG2	green	green
40	SmLS2	green	green
41	SmLS	green	green
42	SmLG3	green	green
43	SmLF4	green	green
44	SmLC1	green	green
45	SmLC8	green	green
46	SmLC10	green	green
47	SmLC05	green	green
48	SmLC91	green	green
49	SmLC92	green	green
50	SmLS167	green	green
51	SmLF1	green	green
52	SmLK1	green	green
53	SmL156	green	green
54	SmL158	green	green
55	SmLQ1	green	green
56	SmLQ2	green	green
57	SmLQ7	green	green
58	SmLQ8	green	green
59	SmLGX1	green	green
60	SmLG83	green	green
61	SmLG79	green	green
62	SmL21F	green	green
63	SmL42	green	green
64	SmLV06	green	green
65	SmLZ31	green	green
66	SmLZ1A	green	green
67	SmB145	white	green
68	SmB112	white	green
69	SmB84	white	white
70	SmB89	white	white
71	SmB21	white	white
72	SmBE9	white	white
73	SmBE14	white	white
74	SmB18	white	white
75	SmBE218	white	white
76	SmBE432	white	white
77	SmBE209	white	white
78	SmBE465	white	white
79	SmB79	white	white
80	SmBE201	white	white
81	SmBE358	white	white
82	SmB31	white	white

83	SmBE359	white	white
84	SmB19	white	white
85	SmBE38	white	white
86	SmB80	white	white
87	SmBE37	white	white
88	SmB197	white	white
89	SmB96	white	white
90	SmB118	white	white
91	SmBE421	white	white
92	SmBE207	white	white
93	SmB222	white	white
94	SmBE39	white	white
95	SmB213	white	white
96	SmBE84	white	white
97	SmB539	white	white
98	SmB540	white	white
99	SmB546	white	white
100	SmB541	white	white
101	SmB545	white	white
102	SmB531	white	white
103	SmB061	white	white
104	SmBG40	white	white
105	SmB326	white	white
106	SmB223	white	white
107	SmB125	white	white
108	SmB235	white	white
109	SmB079	white	white
110	SmB166	white	white
111	SmB176	white	white
112	SmB519	white	white
113	SmBY10	white	white

Table S4 Sequence alignment of BL01 and B1

Type	Sequence
CDS Sequence of BL01	ATGATTTGCATTGAGGATGAATTATTGGGTTGGAAAGATTTCCCAA<br>AGGGGCTTAAAGTCCTACTTCTTGATGAAGACAGCAATTCTGCTG<br>CTGAGATGAGATCAAGGCTTGAGAAAATGGACTACATAGTTTACT<br>CGTTCTGTAATGAGAGTGAAGCTTTAACCGCAATCTCAAGCAAAT<br>CCGAGGGCTTTCATGTTGCCATTGTGGAGGTAAGTGAAGGCAATA<br>GTGATGGGGTTCTCCGATTTCTTGAAAGTGCCAAAGATCTACCAA<br>CTATAATGACATCAAATATTCATTCTCTTAGCACAATGATGAAGTG<br>TATTGCGCTGGGAGCAGTTGAGTTCCTTCAGAAACCATTGTCAGA<br>TGATAAACTCAAAAATATATGGCAGCATGTGGTTCACAAGGCATT<br>CAATTCTAGAAAGGATGTGTCCAGATCGCTTGATCCGGTAAAAGA

	ATCTCTCCTCTCAATGCTACAGCTAAAACCAGCAAAGGATGAAGC<br>AGATGACAAAAATTCAAATCGAATAGAACCTCTCACTGCAATTGC<br>GGAAAGCAACACCGAACAGTTATCGGGCTGTGATAAATACCCTG<br>CTCCCTCAACCCCACAATTGAAACAAGGAGTGCGGTCGGTGGAT<br>GATGGTGATTGCCATGATCATACTATCTTCTCAACTGACCAAGACA<br>GTGGTGAGCATGATGGTGACACTAAATCTGTCGAAACTACATATA<br>ACAATTCACTTGCTGAGAATACTGTCCAAACAAGTCCTCCTGGAC<br>AACAAGGAGAGAGAATCTTGAAAGAGGAGAATGTTTCATCTCCT<br>CATCAAAAGATGGAGGCTAATATTGCTACCTCTTCTCAAAGTAATG<br>ACTGCCCTGACAGTAGCATTAGTCATTCTGCGGAACCTAGTAAAG<br>CATCTGGTCCTCATAGTTCAAGTGGGACTAAATCCAATAAGAAGA<br>AGTTGAAGGTAGATTGGACACCTGAACTACACAAGAAGTTCGTT<br>CAAGCAGTAGAGCAACTCGGTATAGATCAAGCCATTCCTTCTCGA<br>ATACTGGACCTGATGAAAGTAGAGGGCTTAACAAGACATAATGTA<br>GCTAGCCATCTCCAGAAATACAGAATGCATCGGCGGCAACTTTTG<br>CCAAAGGAAGTGGAAAGGAGATGGCCTCATCCACAACCTAGAGA<br>TTCAGTACAAAGGAGTTACTATCCTCATAAACCTATCATGACGTTC<br>CCACCTTATCATTCTAATCATGTCGCCCCAGGTGGTCAATTTTGTC<br>CTGGTTGGTTACCACCAGCAAGTTGTCCGAATGGTTTACAAGTAT<br>GGGGTTCACCATACTATCTGGGATGGAAGCCTGCAGAAACTTGGC<br>ACTGGACTCCTCAACCAGGGCTACATGCTGATACATGGGGCTCCC<br>CTGTCATGGCGTCATCATTTGGATCATATCCACCATATCCTCAGAAT<br>GCTGGAGTGTACCAGCCTCACGGAATGCATAGCAGATATAGCATG<br>CTAGAGAAGTCGTTTGATCTTCACCCGACAGAGGAGGTGATTGAT<br>AAAGTAGTAAAGGAGGCAATAACCAAACCATGGTTACCACTTCC<br>GTTGGGCCTAAAACCTCCTTCCACGGAGGGCGTTCTCGACGAAC<br>TTTCTAGACAAGGGATCTCAACCGTCCCTCCACAAATCAATTGTC<br>GGAGATGA
CDS Sequence of B1	ATGATTTGCATTGAGGATGAATTATTGGGTTGGAAAGATTTCCCAA<br>GGGGGCTTAAAGTCCTACTTCTTGATGAAGACAGCAATTCTGCTG<br>CTGAGATGAGATCAAGGCTTGAGAAAATGGACTACATAGTTTACT<br>CGTTCTGTAATGAGAGTGAAGCTTTAACCGCAATCTCAAGCAAAT<br>CCGAGGGCTTTCATGTTGCCATTGTGGAGGTAAGTGAAGGCAATA<br>GTGATGGGGTTCTCCGATTTCTTGAAAGTGCCAAAGATCTACCAA<br>CTATAATGACATCAAATATTCATTCTCTTAGCACAATGATGAAGTG<br>TATTGCGCTGGGAGCAGTTGAGTTCCTTCAGAAACCATTGTCAGA<br>TGATAAACTCAAAAATATATGGCAGCATGTGGTTCACAAGGCATT<br>CAATTCTAGAAAGGATGTGTCCAGATCGCTTGATCCGGTAAAAGA<br>ATCTCTCCTCTCAATGCTACAGCTAAAACCAGCAAAGGATGAAGC<br>AGATGACAAAAATTCAAATCGAATAGAACCTCTCACTGCAATTGC<br>GGAAAGCAACACCGAACAGTTATCGGGCTGTGATAAATACCCTG<br>CTCCCTCAACCCCACAATTGAAACAAGGAGTGCGGTCGGTGGAT<br>GATGGTGATTGCCATGATCATACTATCTTCTCAACTGACCAAGACA<br>GTGGTGAGCATGATGGTGACA—TAAATCTGTCGAAACTACATATA

	ACAATTCACTTGCTGAGAATACTGTCCAAACAAGTCCTCCTGGAC<br>AACAAGGAGAGAGAATCTTGAAAGAGGAGAATGTTTCATCTCCT<br>CATCAAAAGATGGAGGCTAATATTGCTACCTCTTCTCAAAGTAATG<br>ACTGCCCTGACAGTAGCATTAGTCATTCTGCGGAACCTAGTAAAG<br>CATCTGGTCCTCATAGTTCAAGTGGGACTAAATCCAATAAGAAGA<br>AGTTGAAGGTAGATTGGACACCTGAACTACACAAGAAGTTCGTT<br>CAAGCAGTAGAGCAACTCGGTATAGATCAAGCCATTCCTTCTCGA<br>ATACTGGACCTGATGAAAGTAGAGGGCTTAACAAGACATAATGTA<br>GCTAGCCATCTCCAGAAATACAGAATGCATCGGCGGCAACTTTTG<br>CCAAAGGAAGTGGAAAGGAGATGGCCTCATCCACAACCTAGAGA<br>TTCAGTACAAAGGAGTTACTATCCTCATAAACCTATCATGACGTTC<br>CCACCTTATCATTCTAATCATGTCGCCCCAGGTGGTCAATTTTGTC<br>CTGGTTGGTTACCACCAGCAAGTTGTCCGAATGGTTTACAAGTAT<br>GGGGTTCACCATACTATCTGGGATGGAAGCCTGCAGAAACTTGGC<br>ACTGGACTCCTCAACCAGGGCTACATGCTGATACATGGGGCTCCC<br>CTGTCATGGCGTCATCATTTGGATCATATCCACCATATCCTCAGAAT<br>GCTGGAGTGTACCAGCCTCACGGAATGCATAGCAGATATAGCATG<br>CTAGAGAAGTCGTTTGATCTTCACCCGACAGAGGAGGTGATTGAT<br>AAAGTAGTAAAGGAGGCAATAACCAAACCATGGTTACCACTTCC<br>GTTGGGCCTAAAACCTCCTTCCACGGAGGGCGTTCTCGATGAACT<br>TTCTAGACAAGGGATCTCAACCGTCCCTCCACAAATCAATTGTCG<br>GAGATGA
Protein sequence of BL01	MICIEDELLGWKDFPKGLKVLLLDEDSNSAAEMRSRLEKMDYIVYS<br>FCNESEALTAISSKSEGFHVAIVEVSEGNSDGVLRFLESAKDLPTIMT<br>SNIHSLSTMMKCIALGAVEFLQKPLSDDKLKNIWQHVVHKAFNSRK<br>DVSRSLDPVKESLLSMLQLKPAKDEADDKNSNRIEPLTAIAESNTEQ<br>LSGCDKYPAPSTPQLKQGVRSVDDGDCHDHTIFSTDQDSGEHDGDT<br>KSVETTYNNSLAENTVQTSPPGQQGERILKEENVSSPHQKMEANIAT<br>SSQSNDCPDSSISHSAEPSKASGPHSSSGTKSNKKKLKVDWTPELHK<br>KFVQAVEQLGIDQAIPSRILDLMKVEGLTRHNVASHLQKYRMHRRQ<br>LLPKEVERRWPHPQPRDSVQRSYYPHKPIMTFPPYHSNHVAPGGQF<br>CPGWLPPASCPNGLQVWGSPYYLGWKPAETWHWTPQPGLHADTW<br>GSPVMASSFGSYPPYPQNAGVYQPHGMHSRYSMLEKSFDLHPTEE<br>VIDKVVKEAITKPWLPLPLGLKPPSTEGVLDELSRQGISTVPPQINCR<br>R*
Protein sequence of B1	MICIEDELLGWKDFPRGLKVLLLDEDSNSAAEMRSRLEKMDYIVYS<br>FCNESEALTAISSKSEGFHVAIVEVSEGNSDGVLRFLESAKDLPTIMT<br>SNIHSLSTMMKCIALGAVEFLQKPLSDDKLKNIWQHVVHKAFNSRK<br>DVSRSLDPVKESLLSMLQLKPAKDEADDKNSNRIEPLTAIAESNTEQ<br>LSGCDKYPAPSTPQLKQGVRSVDDGDCHDHTIFSTDQDSGEHDGDI<br>NLSKLHITIHLLRILSKQVLLDNKERES*
